# Changes in diet quality across life transitions from adolescence to early adulthood: a latent growth analysis

**DOI:** 10.1016/j.ajcnut.2024.08.017

**Published:** 2024-09-26

**Authors:** Yinhua Tao, Melanie Wall, Nicole Larson, Dianne Neumark-Sztainer, Eleanor M Winpenny

**Affiliations:** 1 MRC Epidemiology Unit, University of Cambridge, Cambridge, United Kingdom; 2 Department of Psychiatry, Mailman School of Public Health, Columbia University, NY, United States; 3 Division of Epidemiology and Community Health, School of Public Health, University of Minnesota, Minneapolis, MN, United States; 4 Mohn Centre for Children’s Health and Wellbeing, School of Public Health, Imperial College London, London, United Kingdom

**Keywords:** diet, young adult, sex, life events, education, employment, cohabitation, childbirth, longitudinal, the United States

## Abstract

**Background:**

Adolescence to early adulthood is a period of multiple life transitions. These transitions, along with changing resources and contexts, could contribute to significant changes in diet, which may persist into later adulthood.

**Objectives:**

We investigated diet quality trajectories from age 15 to 31 y and changes in diet quality associated with life transitions by sex.

**Methods:**

Data from the Project EAT (Eating and Activity in Teens and Young Adults) study in Minnesota, the United States were used to examine diet quality among a longitudinal cohort (*n* = 2524) across 4 waves (mean ages of 15, 19, 25, and 31 y). Average within-person changes in DASH (Dietary Approaches to Stop Hypertension) scores were analyzed using sex-specific latent growth models, incorporating underlying growth trajectories, 5 life transitions, and baseline sociodemographic and health characteristics.

**Results:**

Both sexes followed a quadratic trajectory of DASH scores, showing decreases in diet quality from Wave 1 to 2 followed by increases until Wave 4. However, males had increasingly worse diet quality than females. Compared with no such transition, leaving the parental home between Waves 1 and 2, was associated with transient decreases in diet quality at Wave 2 only for males (β: −2.34; 95% confidence interval [CI]: −3.57, −1.11). For females, cohabitating with a partner and becoming a parent between Waves 3 and 4 were related to decreases (β: −1.96; 95% CI: −3.45, −0.47) and increases (β: 1.85; 95% CI: 0.47, 3.23), respectively, in diet quality at Wave 4. Leaving full-time education and starting full-time employment showed negative and positive associations, respectively, with long-term diet quality for both sexes.

**Conclusions:**

Diet quality remained suboptimal throughout adolescence and improved across early adulthood. Targeted dietary interventions are welcome for young people who leave their parental home early or do not enter a structured school or workplace environment and for addressing sex differences in diet quality associated with family-related life transitions.

## Introduction

Poor diet quality is a key modifiable risk factor for chronic disease, including diabetes, cardiovascular disease, and other noncommunicable diseases (NCDs) [1]. A systematic analysis of diet-related health risks in 195 countries found that suboptimal diet, particularly high sodium intake and low fruit and whole grain intakes, contributed to substantial NCD mortality and morbidity burdens among adults aged 25 y and above [2]. However, once individuals enter adult life, dietary patterns are fairly stable and tend to track over time [3,4]. The developmental stage of early adulthood may, therefore, represent a window of opportunity for the development of a high-quality diet to prevent NCDs in later life.

Early adulthood (age 16–30 y) is a period of multiple life transitions, which provide an opportunity to disrupt poor eating habits developed during adolescence and establish healthy dietary patterns persisting into adulthood [5,6]. Alongside rapid physiologic and psychologic development [7], young people will be exposed to different food-related environments following early adulthood transitions. Specifically, most young people move out of the parental home, which underlies significant changes in the home food environment and allows them more autonomy in food choices [8,9]. They also complete high school education and choose different education/occupational paths, such as continuing further education or starting a first job, leading to changing exposures to institutional food environments. Besides, these life changes are often accompanied with changes in social environment as young people are exposed to a wider social network, develop partner relationships, and may become a parent. A wide range of exposures within these food-related environments are established correlates of quality of diet [10].

There is emerging longitudinal evidence of changes in diet from adolescence to early adulthood. However, these longitudinal studies are not consistent in their approaches to assessing the timing and duration of dietary changes. Studies in the United States and Australia showed that diet quality declined or stayed low from age 15 y to the early twenties [[11], [12], [13]]. Winpenny et al. [14,15] observed a quadratic trajectory in food group consumption for American and Norwegian young people, indicating decreases in diet quality in their early twenties followed by positive changes until the early thirties. Moreover, the timing of early adulthood transitions may be relevant to changes in dietary intake. For example, adolescents who leave full-time education at a younger age (for example, after high school) are often limited in financial resources and exposed to strong peer networks [13,16], which could lead to significant changes in diet. However, it is not clear whether dietary changes associated with life transitions will persist over time. Compared with transient changes in diet following these transitions, persistent dietary changes are of more concern for public health researchers and practitioners because tailored interventions are required to prevent unhealthy dietary intake patterns from being carried into adulthood. To address these considerations, longitudinal research needs to consider the timing of life transitions and differentiate their transient or persistent associations with changes in diet quality, superimposed on underlying dietary trajectories from adolescence to early adulthood.

This study aims to investigate changes in diet quality and the associations with life transitions from adolescence to early adulthood by sex. To achieve this aim, we analyzed longitudinal cohort data from the Project EAT (Eating and Activity in Teens and Young Adults) study to address the following research questions:

1) How does diet quality change from adolescence to early adulthood (age 15–31 y)?
2) How are education-, employment- and family-related life transitions associated with changes in diet quality?
3) How do changes in diet quality and associations with life transitions differ by sex?

## Methods

### Study design and sample

This study used data from the Project EAT study, a longitudinal investigation of young people’s eating, activity, and weight-related health behaviors [17]. The first wave (Wave 1) of data was collected in 1998–1999. Participants (mean age 14.9; *n* = 4746) from 31 public secondary schools in the Minneapolis–St Paul metropolitan area of Minnesota completed the baseline survey, including sociodemographic and health characteristics, family- and education/employment-related life circumstances, and daily food intake. Follow-up surveys were conducted every 5 y in 2003–2004 (Wave 2, mean age = 19.4; *n* = 2516), 2008–2009 (Wave 3, mean age = 25.3; *n* = 2287), and 2015–2016 (Wave 4, mean age = 31.1; *n* = 1830). For the current analysis, longitudinal participants who completed food frequency questionnaires (FFQ) at 2 or more waves (*n* = 2524) were included. This approach allowed us to retain the maximum number of study participants, while ensuring that included participants reported sufficient data to investigate changes in diet across life transitions (see the flow chart for sample selection in Supplemental Figure 1). Ethical approval for Project EAT was obtained from the University of Minnesota’s Institutional Review Board Human Subjects Committee (1207S17861). Parental consent and written assent from participants were obtained at Wave 1. For each follow-up survey wave, participants reviewed a consent form, and completion of the follow-up survey implied written consent.

### Dietary intake quality

Dietary intake was measured using 2 age-appropriate semiquantitative FFQs. The 1995 version of the Youth and Adolescent Questionnaire was used to assess dietary intake at Wave 1 and Wave 2, and the 2007 grid form of the Willet Adult FFQ was used at Wave 3 and Wave 4 [18,19]. The reproducibility, validity, and comparability of the 2 forms have been described previously [[20], [21], [22]]. For both semiquantitative FFQs, participants reported the frequency of food consumption with reference to specified serving sizes for each food item. The main difference between the 2 FFQs is that the adolescent form of the FFQ included fewer listed items than the adult FFQ (127 compared with 151 food items). However, participants could additionally report intake of foods not listed in the FFQs [22]. To address the consistency of measures of dietary intake over age, we only included in our analysis food items reported across both adolescent and adult FFQs (see Supplemental Table 1) to allow for assessment of longitudinal changes in diet quality.

The DASH (Dietary Approaches to Stop Hypertension) index was used to assess overall diet quality [23]. This index is based on adherence to a DASH diet, which reduces blood pressure and other cardiovascular disease risk factors in clinical trials [24,25]. The DASH diet recommends a healthy dietary pattern rich in the components of fruit, vegetables, whole grains, low-fat dairy, nuts and legumes, and low in red and processed meat, added sugar, and sodium. For each component, a score of 0–10 was assigned based on recommended daily servings [26]. The DASH index was subsequently calculated by summing the scores of 8 components, resulting in a score on a 0–80 scale (0 being the worst and 80 being the best diet quality). Compared with a measure of specific nutrient or food group intake, the DASH index provides a comprehensive assessment of overall diet quality, considering the combination of interrelated nutrients and foods [27].

The included food and beverage items for each DASH component and the method for calculation of the DASH index are provided in Supplemental Table 1. Note that before calculating the DASH index, we excluded the dietary records with implausible energy intakes (<500 or >5000 kcal/d) [22]. To assess diet quality independent of reported total dietary intakes, we uniformly adjusted energy intakes to 2000 kcal (8.37 MJ)/d for both sexes using the residual method [28].

### Life transitions

Five life transitions that are common across early adulthood have been identified using survey measures and previously described in related research [15]. The transitions included in the analysis are *1*) leaving the parental home, *2*) leaving full-time education, *3*) beginning full-time employment, *4*) cohabitating with a partner, and *5*) becoming a parent. These transition variables were assessed by comparing participants’ life circumstances between pairs of waves. The survey questions on life circumstances at each wave were about living arrangements (“During the past year, with whom did you live the majority of the time?”), education status (“Which of the following best describes your student status?”), employment status (“How many hours a week do you work for pay?”), and having children (“How many children do you have?”). For missing data on wave-specific life circumstances, we assumed that life circumstances did not change and thus imputed the data from the previous wave. The transition variables were set as 0 before a transition taking place, and 1 following a transition. Considering our interest in life transitions occurring for the first time, we regarded wave-specific life circumstances which returned to the pretransition state as missing. We thus removed these wave-specific data from the analysis (<10% of the participants for each exposure). For example, if a participant left the parental home between Waves 1 and 2 and then moved back in together with parents at Wave 3, the transition variable of leaving the parental home was set as 0 at Wave 1, 1 at Wave 2, and as missing at Waves 3 and 4.

### Sociodemographic characteristics

We included baseline sociodemographic and health characteristics as covariates in our analysis. Note that sex, self-identified as male or female by participants, was not included as a covariate because our analyses were stratified by sex (as elaborated below). The baseline age in years was adjusted for because of the variance in the age of participants when they were recruited at Wave 1 (ranging from 11 to 18 y). Participants indicated their ethnicity by selecting ≥1 of the following categories: White, Black or African American, Hispanic or Latino, Asian American, Hawaiian or Pacific Islander, or American Indian. Considering the small sample sizes for persons identifying as Hawaiian or Pacific Islander, American Indian, and mixed groups, we combined them into 1 category, termed "other/mixed ethnic groups," in the analysis. Parental socioeconomic status was a 5-level composite index, estimated based on parental education levels but also accounting for family eligibility for public assistance, eligibility for free or reduced-cost school meals, and parental employment status [29]. Baseline health characteristics included self-reported measures of health conditions and general health status. Health conditions were reported in response to the question: “Do you have a physical or health condition that makes it hard for you to do some things other kids your age do, such as concentrating in school, doing sports, or eating like other teenagers?” General health status was self-rated across 4 response categories: poor, fair, good, and excellent. We did not include changes in income, exposure to geographic context, and other health-related behaviors as covariates because these are unlikely to confound the associations but could act as mediators on the pathway between life transitions and changes in diet outcomes.

### Statistical analyses

Statistical analyses were conducted in R (version 4.3.1, analysis code available on the Open Science Framework, https://osf.io/3gvht/). We compared baseline sociodemographic and health characteristics of our study participants with those included in the Project EAT baseline survey, and assessed the occurrence of life transitions and changes in DASH scores across 4 waves. Independent *t*-tests were used to examine sex differences in DASH scores and 8 DASH components at each wave.

Multiple-group latent growth models were used to estimate the trajectories of DASH scores with age and the associations with life transitions by sex. Before testing sex differences in diet, we constructed the population latent growth model (not differentiated by sex) step by step. First, an unconditional growth model was built to fit the population growth curve of DASH scores, showing that including a random intercept, random linear slope, and random quadratic slope produced the best model fit (Supplemental Table 2; Model 1). Given the recruitment of participants from schools, we tested the school-level variances in DASH scores by calculating intraclass correlation coefficients (ICCs). The results show that ICCs were low (3.76% of total variances) at the school level, suggesting no serious issue from sample clustering by school [30], so we did not consider school-level clustering in our analyses. We next added the baseline covariates as predictors of the 3 latent parameters.

A final step was the inclusion of life transition variables to test for deviations in the population growth curve after the transitions. We specified 2 different models to investigate transient and persistent changes in diet quality associated with life transitions. The transient associations were examined by linking each life transition between a pair of waves to the observed DASH score in the following wave (Model 2). The persistent associations were analyzed by relating each life transition between a pair of waves to an additional latent intercept of DASH scores across the following waves (Model 3), interpreted as an overall shift to the underlying growth trajectory [15,31]. To reach a general understanding of the associations between life transitions and diet quality, we also fixed each life transition’s coefficients to be the same across 4 waves in the persistent associations model (Model 4). Note that when estimating the associations of the growth parameters with each life transition, we mutually adjusted for the other 4 life transitions in the models.

**FIGURE 1:**
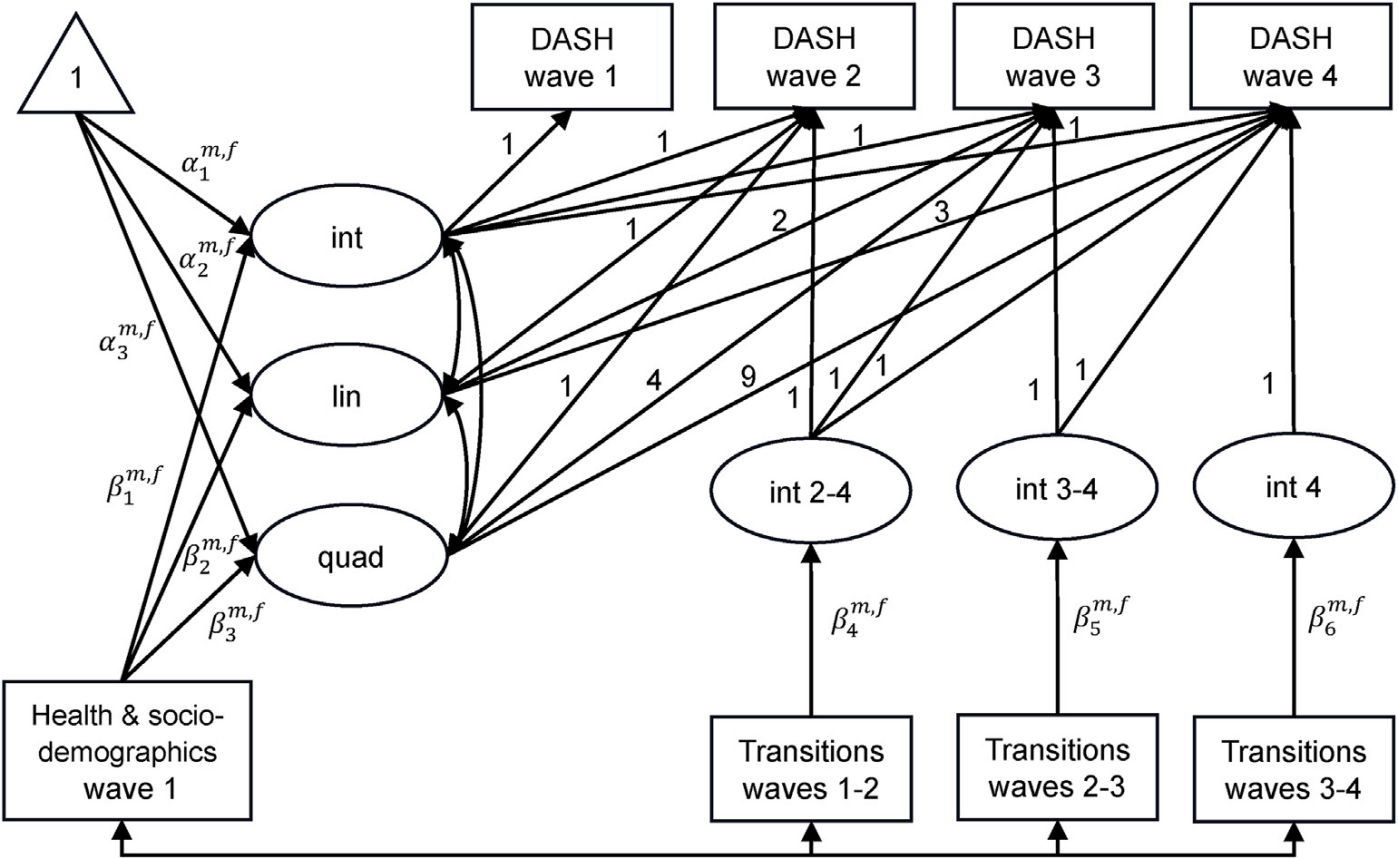
Path diagram of sex-specific latent growth models to investigate associations of life transitions with persistent changes in diet quality after controlling for underlying growth trajectories and baseline socio-demographic and health characteristics. Rectangles, ellipses, and triangles, respectively, represent observed variables, latent variables, and estimates of means. Int 2–4, int 3–4, and int 4 indicate additional intercepts between Waves 2 and 4, between Waves 3 and 4, and at Wave 4, respectively. The numbers on each arrow show the loadings of the latent variables on the observed variables. α and β represent the coefficients, which are freely estimated between m (males) and f (females). DASH, Dietary Approaches to Stop Hypertension; Int, intercept; lin, linear slope; quad, quadratic slope.

After fitting the population growth curve, we tested sex differences in dietary changes in a series of models. This test found that a model where both means and regression parameters varied by sex provided the best fit (Supplemental Tables 3 and 4); that is, males and females followed different growth trajectories of diet quality, and the associations of life transitions and covariates with diet quality were also sex-specific. Figure 1 demonstrates the path diagram of sex-specific latent growth models to investigate associations of life transitions with persistent changes in diet quality (Model 3). The path diagram of the transient associations model (Model 2) is provided in Supplemental Figure 2. Throughout the modeling analysis, the maximum likelihood estimator with robust standard errors was used to account for the non-normal distribution of DASH scores. Missing data were addressed by using full information maximum likelihood estimation. The goodness of fit of the models was assessed based on the comparative fit index, Tucker–Lewis index , root mean square error of approximation, standardized root mean square residual, and the likelihood-based fit indices.

## Results

### Participants’ sociodemographic characteristics

A total of 2524 longitudinal participants who completed FFQs at 2 or more waves were included in our analyses. Research participants had a mean age of 14.9 y (SD = 1.6), 53.9% self-identified as female, and 11.4% reported health conditions at baseline. Compared with the Project EAT participants recruited at Wave 1, our longitudinal research participants included a higher proportion of persons identifying as White (63.0% compared with 47.7%), a lower proportion of persons identifying as Black or African American (8.9% compared with 18.7%), and more participants whose parents were of high socioeconomic status (Supplemental Table 5).

### Prevalence and timing of life transitions

Across 4 survey waves, many research participants experienced each of the 5 life transitions for the first time between ages 15 and 31 y, ranging from 39.8% of participants becoming a parent to 85.5% leaving full-time education (Table 1). Regarding the timing of each life transition, most participants left their parental home between mean ages 15 and 25 y (32.8% of participants between Waves 1 and 2 and 30.3% between Waves 2 and 3). Leaving full-time education (44.3%) and beginning full-time employment (36.8%) occurred most often between Waves 2 and 3, while cohabitation with a partner (18.8%), and especially becoming a parent (19.2%), take place most frequently in the late twenties between Waves 3 and 4. There were few sex differences in the timing and prevalence of life transitions across early adulthood (Supplemental Table 6). Another notable feature was the parallel changes in life circumstances between pairs of waves. For example, over 60% of participants who left full-time education between Waves 1 and 2 (or between Waves 2 and 3) also began full-time employment in the same period (Table 1).

**TABLE 1:** Prevalence and timing of life transitions among research participants (*n* = 2524).

Life transitions	Another life transition occurring in the same wave	Waves 1–2, *n* (%)	Waves 2–3, *n* (%)	Waves 3–4, *n* (%)
Leaving the parental home	*n*	830	765	373
	Leaving full-time education	284 (34.2)	434 (56.7)	106 (28.4)
	Beginning full-time employment	380 (45.8)	385 (50.3)	109 (29.2)
	Cohabitating with a partner	198 (23.9)	403 (52.7)	166 (44.5)
	Becoming a parent	104 (12.5)	164 (21.4)	87 (23.3)
Leaving full-time education	*n*	663	1120	376
	Leaving the parental home	284 (42.8)	434 (38.8)	106 (28.2)
	Beginning full-time employment	429 (64.7)	679 (60.6)	168 (44.7)
	Cohabitating with a partner	194 (29.3)	520 (46.4)	162 (43.1)
	Becoming a parent	158 (23.8)	228 (20.4)	122 (32.4)
Beginning full-time employment	*n*	707	929	315
	Leaving the parental home	380 (53.7)	385 (41.4)	109 (43.6)
	Leaving full-time education	429 (60.7)	669 (72.0)	168 (53.3)
	Cohabitating with a partner	151 (21.4)	411 (44.2)	127 (40.3)
	Becoming a parent	93 (13.2)	162 (17.4)	68 (21.6)
Cohabitating with a partner	*n*	269	828	474
	Leaving the parental home	198 (73.6)	403 (48.7)	166 (35.0)
	Leaving full-time education	194 (72.1)	520 (62.8)	162 (34.2)
	Beginning full-time employment	151 (56.1)	411 (49.6)	127 (26.8)
	Becoming a parent	104 (38.7)	245 (29.6)	181 (38.2)
Becoming a parent	*n*	240	382	484
	Leaving the parental home	104 (43.3)	164 (42.9)	87 (18.0)
	Leaving full-time education	158 (65.8)	228 (59.7)	122 (25.2)
	Beginning full-time employment	93 (38.8)	162 (42.4)	68 (14.0)
	Cohabitating with a partner	104 (43.3)	245 (64.1)	181 (37.4)

### Changes in diet quality over age for males and females

Compared with participants who identified as female, male participants had significantly worse diet quality in terms of overall DASH scores and most DASH component scores across the 4 waves (mean overall scores: 36.6–43.5 for males and 39.7–48.3 for females; Table 2). Exceptions were the components of whole grains, low-fat dairy, and sodium. At Wave 1, males consumed more daily servings of whole grains and low-fat dairy than females. However, sex differences in the intake of these 2 healthy foods were negligible in the following 3 waves. For sodium intake, there was little difference by sex across the 4 waves.

**TABLE 2:** Descriptive statistics of DASH scores and 8 DASH components between Waves 1 and 4 for males (*n* = 1163) and females (*n* = 1361).

		Wave 1	Wave 2	Wave 3	Wave 4
DASH scores, mean (SD), ranging from 0 (the worst) to 80 (the best diet quality)	Males	38.1 (9.4)	36.6 (9.6)	40.0 (11.8)	43.5 (11.8)
	Females	40.2 (9.3)	39.7 (9.6)	44.2 (11.6)	48.3 (11.0)
	*P* value	<0.001	<0.001	<0.001	<0.001
DASH components (servings/d, s/d)					
Fruit, mean (SD), ≥4 s/d recommended	Males	2.2 (2.0)	1.8 (1.5)	1.5 (1.3)	1.6 (1.2)
	Females	2.8 (2.1)	2.2 (1.5)	1.9 (1.6)	2.1 (1.7)
	*P* value	<0.001	<0.001	<0.001	<0.001
Vegetables, mean (SD), ≥4 s/d recommended	Males	1.2 (1.1)	1.2 (1.0)	1.6 (1.5)	2.2 (1.8)
	Females	1.6 (1.2)	1.7 (1.2)	2.2 (1.6)	2.8 (1.9)
	*P* value	<0.001	<0.001	<0.001	<0.001
Nuts and legumes, mean (SD), ≥4 s/d recommended	Males	0.2 (0.3)	0.2 (0.4)	0.3 (0.5)	0.5 (0.6)
	Females	0.3 (0.4)	0.3 (0.4)	0.4 (0.4)	0.5 (0.5)
	*P* value	0.008	0.009	0.01	0.22
Whole grains, mean (SD), ≥3 s/d recommended	Males	1.0 (1.2)	1.0 (1.1)	1.1 (1.3)	0.9 (0.9)
	Females	0.8 (0.9)	1.0 (0.9)	1.1 (1.0)	0.9 (0.7)
	*P* value	<0.001	0.83	0.92	0.99
Low-fat dairy, mean (SD), ≥2 s/d recommended	Males	2.3 (1.8)	2.1 (1.6)	1.8 (1.6)	1.5 (1.3)
	Females	2.1 (1.6)	2.0 (1.3)	1.7 (1.4)	1.5 (1.2)
	*P* value	<0.001	0.03	0.66	0.84
Sodium, mean (SD), ≤2.27 grams/d recommended	Males	2.3 (0.4)	2.3 (0.4)	2.2 (0.5)	2.2 (0.5)
	Females	2.3 (0.4)	2.4 (0.4)	2.2 (0.5)	2.1 (0.5)
	*P* value	0.78	0.18	0.79	0.12
Red and processed meat, mean (SD), ≤2 s/d recommended	Males	0.5 (0.4)	0.5 (0.4)	0.7 (0.6)	0.7 (0.5)
	Females	0.4 (0.3)	0.4 (0.3)	0.6 (0.5)	0.6 (0.5)
	*P* value	<0.001	<0.001	<0.001	0.01
Sugar-sweetened beverages, mean (SD), 0 s/d recommended	Males	1.5 (1.3)	1.5 (1.3)	0.9 (1.3)	0.7 (1.0)
	Females	1.3 (1.3)	1.3 (1.2)	0.7 (1.1)	0.7 (0.8)
	*P* value	0.01	<0.001	<0.001	<0.001

Abbreviation: DASH, Dietary Approaches to Stop Hypertension; SD, standard deviation.

*P* values comparing males’ and females’ diet are calculated based on *t* values of the independent *t* test. The recommended daily servings are based on the requirement for the highest DASH score of each food component.

### Growth trajectories of diet quality for males and females

**FIGURE 2:**
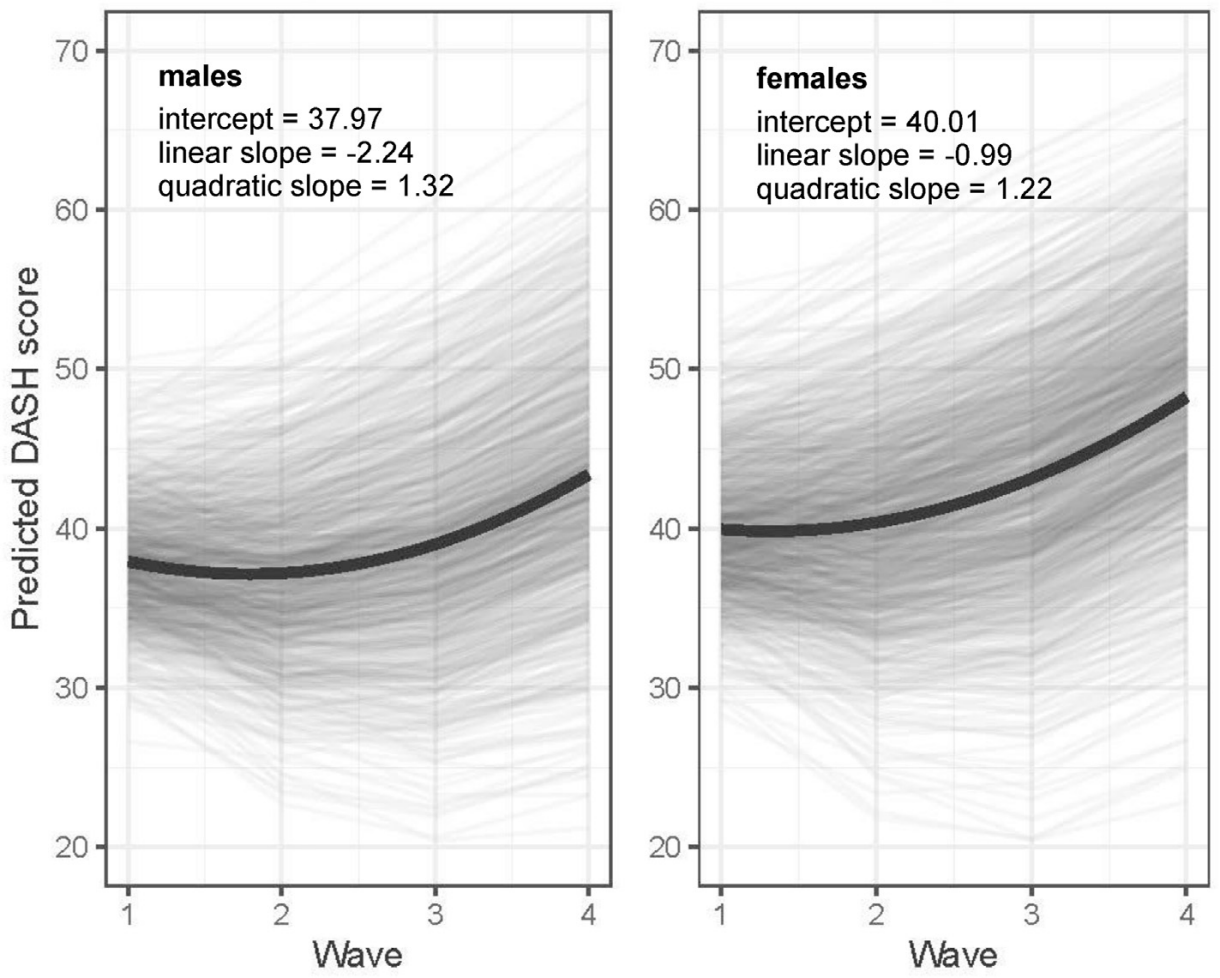
Changes in DASH scores (ranging from 0 to 80, with higher scores representing better diet quality) between Waves 1 and 4 for males and females, predicted from the unconditional growth model (Model 1). The black lines show the latent growth curves of predicted DASH scores, respectively, for males and females, and the gray lines show the predicted DASH scores across 4 waves for each research participant. DASH, Dietary Approaches to Stop Hypertension.

Figure 2 illustrates the latent growth curves of changes in DASH score from Wave 1 to 4 for males and females, respectively, based on results from the unconditional growth model (Model 1). The curves for both sexes followed quadratic growth trajectories: predicted DASH scores dropped slightly from Waves 1 to 2, then rose rapidly from Waves 2 to 4 (*P* < 0.001 for 3 latent parameters of males and females). Compared with females, males had lower means for the latent intercept (37.97 compared with 40.01) and linear slope (−2.24 compared with −0.99). This indicated that males’ diet quality started at a lower level at baseline and had a steeper rate of linear decreases across 4 waves, resulting in a greater initial decrease in diet quality between Waves 1 and 2 and smaller subsequent increases across Waves 2–4 compared with females. At Wave 4, therefore, there was a larger sex difference in predicted DASH scores than at Wave 1.

### Associations of life transitions with changes in diet quality for males and females

TABLE 3, TABLE 4 show the associations of life transitions with transient (Model 2) and persistent (Models 3 and 4) changes in DASH scores for both sexes. Results for the relationship between baseline covariates and diet quality trajectories (for Model 4) are provided in Supplemental Table 7. All associations between a life transition and changes in DASH scores should be interpreted as additional to the underlying growth curve of DASH scores and the influence of other life transitions. Leaving the parental home between Waves 1 and 2 was associated with a transient decrease in DASH scores at Wave 2 only for males (β: −2.34; 95% confidence interval [CI]: −3.57, −1.11, Model 2). However, the negative dietary effect of leaving the parental home between Waves 1 and 2 did not persist over time based on its association with the latent intercept of DASH scores across the following 3 waves (β: −1.05; 95% CI: −2.36, 0.27, Model 3).

**TABLE 3:** Associations of life transitions with transient changes in DASH scores at the subsequent wave for males and females.

	Observed variables of DASH scores at each wave, β (95% confidence interval)					
	Model 2					
	Males			Females		
Life transitions since previous wave	Wave 2	Wave 3	Wave 4	Wave 2	Wave 3	Wave 4
Leaving the parental home	−2.34 (−3.57, −1.11)	0.68 (−0.68, 2.05)	−0.80 (−2.79, 1.18)	−0.65 (−1.64, 0.35)	0.93 (−0.21, 2.07)	−1.43 (−3.04, 0.18)
Leaving full-time education	−2.20 (−3.57, −0.83)	−0.37 (−1.75, 1.01)	0.54 (−1.51, 2.59)	−1.37 (−2.64, −0.11)	−0.82 (−1.99, 0.35)	0.66 (−0.92, 2.24)
Beginning full-time employment	0.81 (−0.54, 2.16)	0.94 (−0.48, 2.37)	0.75 (−1.55, 3.04)	−0.68 (−1.83, 0.47)	1.88 (0.71, 3.05)	0.68 (−0.90, 2.26)
Cohabitating with a partner	0.62 (−1.55, 2.78)	0.63 (−0.97, 2.23)	0.76 (−0.96, 2.47)	−1.17 (−2.57, 0.24)	−0.15 (−1.33, 1.04)	−1.52 (−3.01, −0.03)
Becoming a parent	0.14 (−2.28, 2.56)	−1.65 (−3.83, 0.52)	0.56 (−1.24, 2.35)	1.47 (−0.09, 3.04)	−0.88 (−2.43, 0.66)	1.83 (0.47, 3.20)

Abbreviations: CFI, comparative fit index; DASH, Dietary Approaches to Stop Hypertension; TLI, Tucker–Lewis index; RMSEA, root mean square error of approximation; SRMR, standardized root mean square residual.

Models are adjusted for baseline sociodemographic and health covariates and the underlying growth curve and are mutually adjusted for other life transitions. Model fit indices for Model 2: CFI = 0.978, TLI = 0.961, RMSEA = 0.026, and SRMR = 0.039.

**TABLE 4:** Associations of life transitions with persistent changes in DASH scores across all following waves for males and females.

	Latent intercepts of DASH scores across waves, β (95% CI)							
	Model 3						Model 4	
	Males			Females			Males	Females
Life transitions since previous wave	Wave 2	Wave 3	Wave 4	Wave 2	Wave 3	Wave 4	Across all waves	Across all waves
Leaving the parental home	−1.05 (−2.36, 0.27)	−0.97 (−2.51, 0.56)	−1.21 (−3.28, 0.87)	0.89 (−0.19, 1.97)	0.25 (−1.02, 1.51)	−0.80 (−2.48, 0.90)	−1.07 (−2.17, 0.02)	0.48 (−0.43, 1.40)
Leaving full-time education	−1.97 (−3.39, −0.55)	−2.17 (−3.66, −0.68)	−0.89 (−3.05, 1.27)	−2.35 (−3.59, −1.11)	−1.01 (−2.28, 0.26)	−0.27 (−1.91, 1.37)	−1.86 (−3.00, −0.73)	−1.56 (−2.53, −0.60)
Beginning full-time employment	0.82 (−0.56, 2.20)	1.70 (0.22, 3.18)	1.93 (−0.45, 4.31)	−0.06 (−1.19, 1.08)	0.84 (−0.45, 2.12)	1.70 (0.01, 3.38)	1.27 (0.10, 2.43)	0.49 (−0.46, 1.43)
Cohabitating with a partner	0.74 (−1.52, 3.00)	0.83 (−0.82, 2.49)	1.11 (−0.68, 2.90)	−0.72 (−2.03, 0.60)	−0.51 (−1.70, 0.67)	−1.96 (−3.45, −0.47)	0.88 (−0.37, 2.13)	−0.97 (−1.86, −0.08)
Becoming a parent	0.29 (−1.97, 2.56)	−1.46 (−3.59, 0.68)	0.05 (−1.78, 1.87)	0.55 (−0.90, 1.99)	−0.92 (−2.47, 0.63)	1.85 (0.47, 3.23)	−0.44 (−1.77, 0.89)	0.57 (−0.36, 1.51)

Abbreviations: CFI, comparative fit index; CI, confidence interval; DASH, Dietary Approaches to Stop Hypertension; RMSEA, root mean square error of approximation; SRMR, standardized root mean square residual: TLI, Tucker–Lewis index.

Models are adjusted for baseline sociodemographic and health covariates and the underlying growth curve and are mutually adjusted for other life transitions. Model fit indices for Model 3: CFI = 0.979, TLI = 0.962, RMSEA = 0.026, SRMR = 0.037. Model fit indices for Model 4: CFI = 0.978, TLI = 0.962, RMSEA = 0.026, and SRMR = 0.039.

Across all waves, leaving full-time education was associated with persistent decreases in DASH scores for both sexes (β: −1.86; 95% CI: −3.00, −0.73 for males, and β: −1.56; 95% CI: −2.53, −0.60 for females, Model 4). When separated by wave, these negative associations were stronger when participants left education in earlier waves (Models 2 and 3). In contrast, beginning full-time employment showed an overall positive association with long-term diet quality, particularly among males (β: 1.27; 95% CI: 0.10, 2.43, Model 4). In later waves, beginning employment was associated with a larger increase in DASH scores (Model 3).

Associations of cohabitation and parenthood with diet quality were observed only among females. Across 4 waves, females experienced a persistent decrease in DASH scores after cohabitating with a partner (β: −0.97; 95% CI: −1.86, −0.88, Model 4). Between Waves 3 and 4, particularly, moving in with a partner was related to a great decrease in DASH scores at Wave 4 (β: −1.96; 95% CI: −3.45, −0.47, Model 3), thereby reducing the underlying upward growth trajectory of DASH scores in that period. In contrast, after becoming a parent between Waves 3 and 4, females increased their DASH scores at Wave 4 (β: 1.85; 95% CI: 0.47, 3.23, Model 3).

## Discussion

### Main findings from this study

This is one of the first longitudinal studies to investigate changes in diet quality and associations with life transitions from adolescence to early adulthood, by sex. The results show that diet quality slightly decreased from mid-adolescence to the early twenties and improved until the early thirties for both sexes. Males had worse diet quality scores than females, and this sex difference increased in magnitude across early adulthood. Superimposed on underlying diet quality growth trajectories, life transitions in the education/occupational domains were associated with changes in diet quality. Sex differences in the associations were found for life transitions in the family domain.

### Comparison with previous research

Overall diet quality, measured by the DASH index in this study, followed a quadratic growth trajectory from age 15 to 31 y. The observed low diet quality (mean DASH score of 39.2) between ages 15 and 20 y corresponds to the findings for adolescents’ adherence to a DASH diet in different high-income countries (for example, in the United States [32] and the United Kingdom [33]). Furthermore, a recent systematic review suggested that the DASH diet stayed at a suboptimal level during adolescence with the potential to increase risks of high blood pressure and body mass index gain over the next 10 y [34]. Existing longitudinal evidence on changes in overall diet quality from adolescence to early adulthood has shown a stable unhealthy dietary intake, or increasingly worse diet quality, until the early twenties [9,[11], [12], [13],^35^]. Our study extended the evidence by examining longitudinal changes in diet from adolescence to the early thirties, finding an increase in diet quality after the initial decrease from adolescence to the early twenties.

Longitudinal studies of sex differences in dietary trajectories are mixed in their findings on changes in food group consumption from adolescence to early adulthood. The Norwegian Longitudinal Health Behaviour Study reported that both sexes decreased their intake of fruit and vegetables between ages 14 and 21 y and then increased their intake until the early thirties [14,36]. Meanwhile, the ASH study in the United Kingdom showed that despite similar fruit and vegetable intake between both sexes at age 11 y, females ate more daily servings of fruit and vegetables than males 20 y later [37]. Despite different settings, both of these studies show patterns that correspond to our own findings in this United States-based study of diet quality. To our knowledge, the only evidence of sex-specific growth trajectories of overall diet quality was provided from the Raine Study in Australia, where males were more likely to follow a trajectory of steady increases in Western diet scores, an indicator of unhealthy dietary patterns, than females from ages 14 to 22 y [13]. Moreover, our results, to some extent, explain the established evidence of a less healthy diet for males during adulthood [38], which may develop from their worse diet quality compared with females during adolescence and increasing sex differences across early adulthood.

Few studies have analyzed changes in diet quality across life transitions after taking into account long-term dietary growth trajectories. For the transition of leaving the parental home, our results showed that males decreased diet quality at Wave 2 after moving out of the parental home between Waves 1 and 2 (mean age 15–19 y). This decrease was superimposed on the downward dietary growth trajectory at that time, thus linked to an additional decline in diet quality. Cross-sectional studies on university freshmen also found that compared with living in the family home, living independently was associated with less healthy eating habits and food intake (for example, skipping meals more frequently and consuming fewer daily servings of fruit and vegetables) [39,40], especially among male students [41]. Notably, after specifying the temporal effects of life transitions, our longitudinal analysis indicated that the transient decrease in diet quality after leaving early from the parental home did not persist across early adulthood.

Our findings for the transitions of leaving full-time education and beginning full-time employment suggest that spending time in institutional settings, such as schools and workplaces, was beneficial to diet quality across early adulthood. For education-related transitions, there is well-documented evidence of negative changes in weight-related behaviors (for example, decreases in physical activity and increases in alcohol use) after entering university, contributing to first-semester weight gain [[42], [43], [44]]. In contrast, little attention has been paid to dietary changes for people who leave education after high school [16]. Our study found that leaving full-time education, especially at an early age, had a long-term association with decreases in diet quality persisting into the early thirties. This could result from the loss of accessibility to healthy school meals, especially for high-school students from low-income households.

For occupational transitions, a recent systematic review showed inconsistent results for changes in food consumption after starting employment (increases in fast food intake but no significant changes in the consumption of fruits, vegetables, confectionery fats, and sugar-sweetened beverages) [45]. Our study found that starting a first job showed persistent associations with improvement in diet quality, particularly among males. This association was larger when people began full-time employment later in early adulthood. Possible mechanisms are that workplaces or schools, with a structured day (for example, days with pre-planned and segmented sections) and a proper place for eating and food purchasing (for example, dining halls and healthy food stores), help to avoid poor eating habits and improve diet quality [46]. Active participation in education and employment activities would also increase people’s income in the long term and thus allow for the purchase of more nutrient-dense foods.

Family-related transitions of cohabitation and parenthood were associated with changes in diet quality among females, but not among males. Females decreased their diet quality after beginning cohabitation with a partner during the later period of early adulthood (mean ages 25–31 y). Previous research has found that the associations of cohabitation with diet quality depended on the partner’s diet [37,47,48]. This conforms to our results, considering that males had a worse diet quality than females on average, thereby resulting in a negative impact for cohabitating females and partly canceling out their underlying increases in diet quality in the late twenties. For parenthood, previous longitudinal research did not observe significant changes in parents’ intake of selected food groups, nutrients, and overall diet quality [35,[49], [50], [51]]. After isolating the negative dietary effects of cohabitation from parenthood, our study found that females improved their diet quality after giving birth to a first child in the later period of early adulthood. This could result from fewer constraints from financial and time resources in this period than earlier, so that mothers could pay more attention to healthy diets to benefit their children [52].

### Strengths and limitations of this study

This study used a comprehensive measure of diet, the food frequency questionnaire, which allows for the assessment of habitual diet, total energy intake, and overall diet quality. Project EAT is one of the few studies with longitudinal data on daily intakes of food items and nutrients across early adulthood [5]. In common with other studies spanning adolescence and early adulthood, different FFQs were used across the age range [13,22]. To accommodate this change in FFQ, we only included food items consistently reported across both FFQs in our longitudinal analysis. As with all self-reported measures of diet, these data are subject to participants’ misreporting. Our analyses adjusted for total energy intake to estimate diet quality independently from reported energy intake. Any under- or over-reporting of diet quality was unlikely to influence our findings for within-individual dietary changes, unless there were systematic variations in measurement error with age or across transitions.

This study focused on the association of specific life transitions with deviations from sex-specific dietary growth trajectories, after mutually adjusting for other life transitions. This is a much more robust analysis than other longitudinal studies that have examined dietary changes pre-post a transition [35,43,47,50], without considering other concurrent life changes and the persistence of dietary changes. Our findings, therefore, provide better causal inference for the effect of a single transition on diet quality in the long term. Even so, our within-individual analysis was unable to investigate how different life transitions interact with each other to (re)shape diet quality over time. For example, the negative dietary effect of leaving the parental home after high school might be modified by different education- and employment-related life paths (for example, entering university education compared with starting a first job).

The baseline participants from the Project EAT study and longitudinal participants included in this study were overrepresented for certain ethnic groups (that is, White and Asian American) and people of high socioeconomic status in the metropolitan area of Minnesota. The potential for bias resulting from sample selection (at baseline) and attrition (at follow-ups) is common in longitudinal research on young adults based on results from a systematic review [5]. In this study, the high retention of certain socioeconomically advantaged participants might lead to a general overestimation of diet quality [53,54], and the estimation of within-person changes in diet quality across early adulthood transitions may also be biased toward this population. In addition, data drawing upon a single longitudinal cohort cannot separate dietary growth trajectories over age from secular trends in diet. However, repeated cross-sectional studies using data from the National Health and Nutrition Examination Survey found that population diet quality showed very modest improvement during the study period of Project EAT (1999–2016) [55,56], suggesting that our results indeed reflect age-related changes in diet quality. There is a need for future research to validate the extent to which our findings can be generalized to other population cohorts, underrepresented populations, and socioeconomically deprived areas.

### Conclusions

Adolescence to early adulthood represents a critical life stage when a myriad of early adulthood transitions take place, which may have a persistent impact on diet quality and diet-related health outcomes in later life. The positive changes in diet quality that we observed associated with continued education after high school and beginning employment in the late period of early adulthood could contribute to well-established socioeconomic inequalities in diet across adulthood [53,54]. The results of this study support public health interventions that consider addressing sex differences in dietary changes associated with life transitions in the family domain and target the early adult subgroup who leave the parental home at a young age or do not enter into a structured educational or workplace environment. Given the over-representation of White and Asian Americans and people of high socioeconomic status in this study, we welcome future research to examine the generalizability of our study results to other populations.

## Author contributions

The authors’ responsibilities were as follows – YT: designed the research, performed statistical analysis, and drafted and prepared the manuscript for publication; MW: advised on statistical aspects of the study; NL: provided essential materials and interpreted the results; DN-S: was the principal investigator of the Project EAT study and interpreted the results; EMW: designed the research and interpreted the results; and all authors: read and approved the final manuscript.

## Conflict of interest

The authors report no conflicts of interest.

## Funding

Data collection for the study was supported by grant number R01HL116892 from the National Heart, Lung, and Blood Institute. The authors’ time to conduct and describe the analysis reported within this manuscript was supported by grant number R35HL139853 from the National Heart, Lung, and Blood Institute. The work of Eleanor M. Winpenny and Yinhua Tao was supported by the Medical Research Council MR/T010576/1. The content is solely the responsibility of the authors and does not necessarily represent the official views of the National Heart, Lung, and Blood Institute or the National Institutes of Health.

## Data availability

The datasets generated and/or analyzed during the current study are not publicly available but are available from the senior author (Dianne Neumark-Sztainer, e-mail: neuma011@umn.edu) upon reasonable request.

## References

[1] GBD 2017 Risk Factor Collaborators (2018). Global, regional, and national comparative risk assessment of 84 behavioural, environmental and occupational, and metabolic risks or clusters of risks for 195 countries and territories, 1990–2017: a systematic analysis for the Global Burden of Disease Study 2017. Lancet.

[2] GBD 2017 Diet Collaborators (2019). Health effects of dietary risks in 195 countries, 1990–2017: a systematic analysis for the Global Burden of Disease Study 2017. Lancet.

[3] Craigie A.M., Lake A.A., Kelly S.A., Adamson A.J., Mathers J.C. (2011). Tracking of obesity-related behaviours from childhood to adulthood: a systematic review. Maturitas.

[4] Chong M.F. (2022). Dietary trajectories through the life course: opportunities and challenges. Br. J. Nutr..

[5] Winpenny E.M., Penney T.L., Corder K., White M., van Sluijs E.M. (2017). Change in diet in the period from adolescence to early adulthood: a systematic scoping review of longitudinal studies. Int. J. Behav. Nutr. Phys. Act..

[6] Christoph M.J., Larson N.I., Winkler M.R., Wall M.M., Neumark-Sztainer D. (2019). Longitudinal trajectories and prevalence of meeting dietary guidelines during the transition from adolescence to young adulthood. Am. J. Clin. Nutr..

[7] Viner R.M., Ross D., Hardy R., Kuh D., Power C., Johnson A. (2015). Life course epidemiology: recognising the importance of adolescence. J. Epidemiol. Community. Health.

[8] Shepherd R., Dennison C.M. (1996). Influences on adolescent food choice. Proc. Nutr. Soc..

[9] Cruz F., Ramos E., Lopes C., Araújo J. (2018). Tracking of food and nutrient intake from adolescence into early adulthood. Nutrition.

[10] Sallis J.F., Owen N., Fisher E. (2015). Ecological models of health behavior. Health Behav. Theory Res. Practice..

[11] Lipsky L.M., Haynie D.L., Liu D., Chaurasia A., Gee B., Li K. (2015). Trajectories of eating behaviors in a nationally representative cohort of U.S. adolescents during the transition to young adulthood. Int. J. Behav. Nutr. Phys. Act..

[12] Lipsky L.M., Nansel T.R., Haynie D.L., Liu D., Li K., Pratt C.A. (2017). Diet quality of US adolescents during the transition to adulthood: changes and predictors. Am. J. Clin. Nutr..

[13] Appannah G., Murray K., Trapp G., Dymock M., Oddy W.H., Ambrosini G.L. (2021). Dietary pattern trajectories across adolescence and early adulthood and their associations with childhood and parental factors. Am. J. Clin. Nutr..

[14] Winpenny E.M., van Sluijs E.M., White M., Klepp K.I., Wold B., Lien N. (2018). Changes in diet through adolescence and early adulthood: longitudinal trajectories and association with key life transitions. Int. J. Behav. Nutr. Phys. Act..

[15] Winpenny E.M., Winkler M.R., Stochl J., Van Sluijs E.M., Larson N., Neumark-Sztainer D. (2020). Associations of early adulthood life transitions with changes in fast food intake: a latent trajectory analysis. Int. J. Behav. Nutr. Phys. Act..

[16] Poobalan A.S., Aucott L.S., Clarke A., Smith W.C. (2014). Diet behaviour among young people in transition to adulthood (18–25 year olds): a mixed method study, Health Psychol. Behav. Med..

[17] Neumark-Sztainer D., Wall M.M., Chen C., Larson N.I., Christoph M.J., Sherwood N.E. (2018). Eating, activity, and weight-related problems from adolescence to adulthood. Am. J. Prev. Med..

[18] Feskanich D., Rimm E.B., Giovannucci E.L., Colditz G.A., Stampfer M.J., Litin L.B. (1993). Reproducibility and validity of food intake measurements from a semiquantitative food frequency questionnaire. J. Am. Diet. Assoc..

[19] Rockett H.R., Breitenbach M., Frazier A.L., Witschi J., Wolf A.M., Field A.E. (1997). Validation of a youth/adolescent food frequency questionnaire. Prev. Med..

[20] Rimm E.B., Giovannucci E.L., Stampfer M.J., Colditz G.A., Litin L.B., Willett W.C. (1992). Reproducibility and validity of an expanded self-administered semiquantitative food frequency questionnaire among male health professionals. Am. J. Epidemiol..

[21] Rockett H.R., Wolf A.M., Colditz G.A. (1995). Development and reproducibility of a food frequency questionnaire to assess diets of older children and adolescents. J. Am. Diet. Assoc..

[22] Larson N., Harnack L., Neumark-Sztainer D. (2012). Assessing dietary intake during the transition to adulthood: a comparison of age-appropriate FFQ for youth/adolescents and adults. Public Health Nutr.

[23] Fung T.T., Chiuve S.E., McCullough M.L., Rexrode K.M., Logroscino G., Hu F.B. (2008). Adherence to a DASH-style diet and risk of coronary heart disease and stroke in women. Arch. Intern. Med..

[24] Appel L.J., Moore T.J., Obarzanek E., Vollmer W.M., Svetkey L.P., Sacks F.M. (1997). A clinical trial of the effects of dietary patterns on blood pressure. DASH Collaborative Research Group. N. Engl. J. Med..

[25] Siervo M., Lara J., Chowdhury S., Ashor A., Oggioni C., Mathers J.C. (2015). Effects of the Dietary Approach to Stop Hypertension (DASH) diet on cardiovascular risk factors: a systematic review and meta-analysis. Br. J. Nutr..

[26] Günther A.L., Liese A.D., Bell R.A., Dabelea D., Lawrence J.M., Rodriguez B.L. (2009). Association between the dietary approaches to hypertension diet and hypertension in youth with diabetes mellitus. Hypertension.

[27] Miller P.E., Cross A.J., Subar A.F., Krebs-Smith S.M., Park Y., Powell-Wiley T. (2013). Comparison of 4 established DASH diet indexes: examining associations of index scores and colorectal cancer. Am. J. Clin. Nutr..

[28] Willett W. (2012).

[29] Sherwood N.E., Wall M., Neumark-Sztainer D., Story M. (2009). Effect of socioeconomic status on weight change patterns in adolescents. Prev. Chronic Dis..

[30] Lai M.H., Kwok O.M. (2015). Examining the rule of thumb of not using multilevel modeling: the “design effect smaller than two” rule. J. Exp. Educ..

[31] Curran P.J., Muthén B.O., Harford T.C. (1998). The influence of changes in marital status on developmental trajectories of alcohol use in young adults. J. Stud. Alcohol..

[32] Cohen J.F., Lehnerd M.E., Houser R.F., Rimm E.B. (2017). Dietary approaches to stop hypertension diet, weight status, and blood pressure among children and adolescents: National Health and Nutrition Examination Surveys 2003–2012. J. Acad. Nutr. Diet..

[33] Winpenny E.M., Greenslade S., Corder K., Van Sluijs E.M. (2018). Diet quality through adolescence and early adulthood: cross-sectional associations of the dietary approaches to stop hypertension diet index and component food groups with age. Nutrients.

[34] Paula Bricarello L., Poltronieri F., Fernandes R., Retondario A., de Moraes Trindade E.B.S., de Vasconcelos F. A.G. (2018). Effects of the Dietary Approach to Stop Hypertension (DASH) diet on blood pressure, overweight and obesity in adolescents: a systematic review. Clin. Nutr. ESPEN..

[35] Smith K.J., McNaughton S.A., Gall S.L., Otahal P., Dwyer T., Venn A.J. (2017). Associations between partnering and parenting transitions and dietary habits in young adults. J. Acad. Nutr. Diet..

[36] Lien N., Lytle L.A., Klepp K.I. (2001). Stability in consumption of fruit, vegetables, and sugary foods in a cohort from age 14 to age 21. Prev. Med..

[37] Lake A.A., Adamson A.J., Craigie A.M., Rugg-Gunn A.J., Mathers J.C. (2009). Tracking of dietary intake and factors associated with dietary change from early adolescence to adulthood: the ASH30 study. Obes. Facts..

[38] Imamura F., Micha R., Khatibzadeh S., Fahimi S., Shi P., Powles J. (2015). Dietary quality among men and women in 187 countries in 1990 and 2010: a systematic assessment. Lancet Glob. Health.

[39] Harker D., Sharma B., Harker M., Reinhard K. (2010). Leaving home: food choice behavior of young German adults. J. Bus. Res..

[40] Pengpid S., Peltzer K. (2020). Prevalence and associated factors of skipping breakfast among university students from 28 countries: a cross-sectional study. Int. J. Adolesc. Med. Health.

[41] Van den Bogerd N., Maas J., Seidell J.C., Dijkstra S.C. (2019). Fruit and vegetable intakes, associated characteristics and perceptions of current and future availability in Dutch university students. Public Health Nutr.

[42] Pullman A.W., Masters R.C., Zalot L.C., Carde L.E., Saraiva M.M., Dam Y.Y. (2009). Effect of the transition from high school to university on anthropometric and lifestyle variables in males. Appl. Physiol. Nutr. Metab..

[43] Deforche B., Van Dyck D., Deliens T., De Bourdeaudhuij I. (2015). Changes in weight, physical activity, sedentary behaviour and dietary intake during the transition to higher education: a prospective study. Int. J. Behav. Nutr. Phys. Act..

[44] Hootman K.C., Guertin K.A., Cassano P.A. (2018). Stress and psychological constructs related to eating behavior are associated with anthropometry and body composition in young adults. Appetite.

[45] Winpenny E.M., Smith M., Penney T., Foubister C., Guagliano J.M., Love R. (2020). Changes in physical activity, diet, and body weight across the education and employment transitions of early adulthood: a systematic review and meta-analysis. Obes. Rev..

[46] Zosel K., Monroe C., Hunt E., Laflamme C., Brazendale K., Weaver R.G. (2022). Examining adolescents’ obesogenic behaviors on structured days: a systematic review and meta-analysis. Int. J. Obes. (Lond)..

[47] Berge J.M., MacLehose R., Eisenberg M.E., Laska M.N., Neumark-Sztainer D. (2012). How significant is the ‘significant other’? Associations between significant others’ health behaviors and attitudes and young adults’ health outcomes. Int. J. Behav. Nutr. Phys. Act..

[48] Werneck A.O., Winpenny E.M., Foubister C., Guagliano J.M., Monnickendam A.G., van Sluijs E.M. (2020). Cohabitation and marriage during the transition between adolescence and emerging adulthood: a systematic review of changes in weight-related outcomes, diet and physical activity. Prev. Med. Rep..

[49] Elstgeest L.E., Mishra G.D., Dobson A.J. (2012). Transitions in living arrangements are associated with changes in dietary patterns in young women. J. Nutr..

[50] Laroche H.H., Wallace R.B., Snetselaar L., Hillis S.L., Steffen L.M. (2012). Changes in diet behavior when adults become parents. J. Acad. Nutr. Diet..

[51] Corder K., Winpenny E.M., Foubister C., Guagliano J.M., Hartwig X.M., Love R. (2020). Becoming a parent: a systematic review and meta-analysis of changes in BMI, diet, and physical activity. Obes. Rev..

[52] Burke V., Beilin L.J., Dunbar D., Kevan M. (2004). Changes in health-related behaviours and cardiovascular risk factors in young adults: associations with living with a partner. Prev. Med..

[53] Giskes K., Avendaňo M., Brug J., Kunst A.E. (2010). A systematic review of studies on socioeconomic inequalities in dietary intakes associated with weight gain and overweight/obesity conducted among European adults. Obes. Rev..

[54] Tao Y., Maddock J., Howe L.D., Winpenny E.M. (2024). Early adulthood socioeconomic trajectories contribute to inequalities in adult diet quality, independent of childhood and adulthood socioeconomic position. J. Epidemiol. Commun. H..

[55] Shan Z., Rehm C.D., Rogers G., Ruan M., Wang D.D., Hu F.B. (2019). Trends in dietary carbohydrate, protein, and fat intake and diet quality among US adults, 1999–2016. JAMA.

[56] Liu J., Rehm C.D., Onopa J., Mozaffarian D. (2020). Trends in diet quality among youth in the United States, 1999–2016. JAMA.

